# Amide Proton Transfer-Weighted Magnetic Resonance Imaging for Detecting Severity and Predicting Outcome after Traumatic Brain Injury in Rats

**DOI:** 10.1089/neur.2021.0064

**Published:** 2022-07-15

**Authors:** Yinfeng Dong, Yanting Gu, Jianhua Lu, Jieru Wan, Shanshan Jiang, Raymond C. Koehler, Jian Wang, Jinyuan Zhou

**Affiliations:** ^1^ Department of Anesthesiology and Critical Care Medicine, Department of Radiology, Johns Hopkins University School of Medicine, Baltimore, Maryland, USA.; ^2^ Division of MR Research, Department of Radiology, Johns Hopkins University School of Medicine, Baltimore, Maryland, USA.

**Keywords:** amide proton transfer-weighted imaging, Barnes maze, microglia, MRI, neuroinflammation, traumatic brain injury

## Abstract

After traumatic brain injury (TBI), early assessment of secondary injury severity is critically important for estimating prognosis and treatment stratification. Currently, secondary injury severity is difficult to estimate. The objective of this study was to investigate the capacity of non-invasive amide proton transfer-weighted (APTw) magnetic resonance imaging (MRI) techniques to assess TBI injury in different brain regions and predict long-term neurobehavior outcomes. Fifty-five male and female rats were subjected to a controlled cortical impact with one of three different impactor depths to produce different degrees of TBI. Multi-parameter MRI data were acquired on a 4.7-Tesla scanner at 1 h, 1 day, and 3 days. Immunofluorescence staining was used to detect activated microglia at 3 days, and neurobehavioral tests were performed to assess long-term outcomes after 28 days. The APTw signal in the injury core at 1 day correlated with deficits in sensorimotor function, the sucrose preference test (a test for anhedonia), and spatial memory function on the Barnes maze. The APTw signal in the perilesion ipsilateral cortex gradually increased after TBI, and the value at 3 days correlated with microglia density at 3 days and with spatial memory decline and anhedonia at 28 days. The correlation between APTw and activated microglia was also observed in the ipsilateral thalamus, and its correlation to memory deficit and depression was evident in other ipsilateral sites. These results suggest that APTw imaging can be used for detecting secondary injury and as a potential predictor of long-term outcomes from TBI.

## Introduction

Traditionally, the stages of injury arising from brain trauma are categorized as a primary mechanically induced injury and a delayed secondary injury.^1^ Inflammation is one contributor to secondary injury^1–3^ that continues to evolve long after the initial trauma.^4,5^ Moreover, emotional and cognitive disorders are common long-term complications that may be modulated by neuroinflammation. Developing and implementing early interventions will require noninvasive methods that can stratify the injury severity as early as possible.

Neuroimaging is an important tool in the diagnosis and characterization of traumatic brain injury (TBI) and the prediction of outcome,^6–17^ but more sensitive and reliable techniques would be useful in discerning ongoing pathophysiological processes.^18,19^ A number of pre-clinical and clinical studies have shown that amide proton transfer-weighted (APTw) imaging, a novel chemical exchange saturation transfer magnetic resonance imaging (MRI) technique based on tissue pH and the concentration of endogenous mobile proteins and peptides,^20,21^ could detect brain tumors,^22,23^ brain ischemic injury,^24–26^ and cerebral hemorrhagic injury.^27^ Past studies also have indicated that protein-based APTw MRI can sensitively and non-invasively visualize ischemic damage, inflammatory responses, and several other key pathological processes in TBI, thus improving the capability of MRI to objectively assess TBI.^28,29^ However, TBI outcome depends on injury severity and the particular brain regions that are damaged. Therefore, before APTw MRI can be developed as an early biomarker, work is needed to determine whether it is sensitive to TBI severity and correlates with the degree of long-term neurobehavioral deficits.

In this study, we used multi-parameter MRI, including APTw and several conventional MRI parameters, such as T_2_, T_1_, isotropic apparent diffusion constant (ADC), cerebral blood flow (CBF), and magnetization transfer ratio (MTR), to determine spatial and temporal changes that occur within the first 3 days after a controlled cortical impact (CCI) of different severities in rats. We used immunofluorescence to assess microglial cell density at day 3 post-TBI and neurobehavioral tests over a 1-month recovery period to determine whether the early ATPw signal changes in different regions of interest (ROIs) correlate with the number of microglia and long-term behavioral outcomes.

## Methods

### Animals

Fifty-five adult male (*n* = 29) and female (*n* = 27) Sprague-Dawley rats (240–300 g) were obtained from Charles River Laboratories (Frederick, MD) and acclimated in the Johns Hopkins University vivarium for a minimum of 1 week before surgery. They were randomly divided into four groups: sham surgery group (*n* = 7); mild TBI group (*n* = 16); moderate TBI group (*n* = 17); and severe TBI group (*n* = 16). Of the 49 rats subjected to CCI, 29 survived to the pre-planned survival duration of ∼28 days post-injury (9/10/10 for mild/moderate/severe TBI groups, respectively), 19 were euthanized at the 3-day survival duration for early histological evaluation (7/6/6 for mild/moderate/severe TBI groups, respectively), and 1 was humanely euthanized because it developed severe complications 1 day after TBI. All experimental procedures were approved by the Johns Hopkins University Animal Care and Use Committee and were reported in accordance with the Animal Research: Reporting In Vivo Experiments (ARRIVE) guidelines.

### Controlled cortical impact model of traumatic brain injury

Rats were anesthetized with 5% isoflurane and maintained with 1.5–2.0% isoflurane in oxygen-enriched air. After the heads were secured in a stereotactic frame, the surgical procedure was carried out as described in previous studies.^28,29^ A 4-mm craniotomy was made over the left parietal cortex (bregma: 1 mm posterior, 1 mm lateral). To produce a CCI, the bone flap was removed and the dura was impacted with a benchmark stereotaxic impactor (IM10244; Leica Biosystems, Inc., Richmond, VA) with a diameter of 3 mm at a velocity of 5.5 m/s and a dwell time of 100 ms. To produce a broad range of injury for regression analysis with APTw, impact depths were varied over a wide range (1, 3, or 5 mm). For the purposes of analysis in this study, we refer to the 1-, 3-, and 5-mm depths as mild, moderate, and severe injury, respectively, although it was appreciated that the 5-mm depth would be greater than what is typically used in the rat CCI model. Rats in the sham group were subjected to anesthesia and a scalp incision, but did not undergo a craniotomy or impact. In all groups, the scalp was closed with nylon sutures and antibiotic ointment was applied (Neosporin; Johnson & Johnson, New Brunswick, NJ).

### Magnetic resonance imaging data acquisition and analysis

MRI data were acquired at 1 h, 1 day, 3 days, and 28 days on a 4.7-Tesla (T) horizontal bore animal imager (Bruker Biospin, Billerica, MA) with an actively decoupled cross-coil setup (a 70-mm body coil for radiofrequency transmission and a 25-mm surface coil for signal reception). The multi-parametric MRI protocol used in this study included coronal T_2_w, T_1_w, and T_2_*w sequences: repetition time (TR) = 3/0.7/0.7 sec; echo time (TE) = 64/10/10 ms; seven slices; thickness = 1.5 mm; field of view = 32 × 32 mm^2^; matrix = 192 × 192; and number of averages (NA) = 2/10/10.

Six quantitative or semiquantitative single-slice MRI sequences were acquired, including T_2_ (TR = 3 sec; TE = 30, 40, 50, 60, 70, 80, and 90 ms; NA = 4), T_1_ (inversion recovery; pre-delay = 3 sec; TE = 30 ms; inversion recovery times = 0.5, 0.3, 0.6, 1.2, 1.8, 2.5, and 3.5 sec; NA = 4), isotropic ADC (TR = 3 sec; TE = 80 ms; b-values = 0, 166.7, 333.3, 500, 666.7, 833.3, and 1000 sec/mm^2^; NA = 8), CBF (arterial spin labeling; 3-sec labeling at a distance of 20 mm away from the imaging slice; TR = 6 sec; TE = 28.6 ms), APTw (frequency-labeling offsets of ±3.5 ppm; TR = 10 sec; TE = 30 ms; saturation power = 1.3 μT; saturation time = 4 sec; NA = 16), and MTR (with the same experimental parameters as APTw, except a saturation frequency offset of 10 ppm or 2 kHz at 4.7 T).

Data were processed with Interactive Data Language software (IDL, version 7; Exelis Visual Information Solutions, Inc., Boulder, CO). The T_1_, T_2_, and ADC maps were fitted using I = A+B exp (-TI/T_1_; where A and B are the other two fitting parameters in addition to T_1_), I = I_0_ exp (-TE/T_2_), and I = I_0_ exp (-b ADC), respectively. The CBF map was reconstructed from images with and without labeling, using previously described methods.^30^ APTw images were calculated based on MTR asymmetry at ±3.5 ppm: MTR_asym_(3.5 ppm) = 100% × [S_sat_(-3.5 ppm)/S_0_ – S_sat_(+3.5 ppm)/S_0_], where S_sat_ and S_0_ are the signal intensities with and without selective radiofrequency irradiation, respectively. This definition leads to negative APTw values in the normal brain.^20,21^ The MTR map at 2 kHz was calculated using: 100% × [1 - S_sat_(2 kHz)/S_0_]. The obtained images were interpolated to 384 × 384. Multiple ROIs were drawn manually for quantitative analysis, using MTR and T_2_w as a reference, and then transferred to identical sites on all other coregistered MRI maps. These ROIs were drawn in the same brain locations for all experimental groups. Finally, TBI contusion injury volumes were calculated according to T_2_w hyperintense volumes at 28 days post-TBI.

### Neurological severity score

Investigators blind to group assignments evaluated neurological function on 1, 3, and 28 days post-TBI by a modified neurological severity score (mNSS).^28^ The mNSS included motor (muscle status and abnormal movement), sensory (visual, tactile, and proprioceptive), reflex (pina reflex, corneal reflex, and startle reflex), and balance tests. Function was graded on a scale of 0–18, where 0 is normal and 18 indicates the most severe neurological deficiency.

### Barnes maze

Barnes maze testing is commonly used as an assessment of spatial memory and learning.^31^ During the fourth week of recovery, rats were first subjected to a 4-day training period. For each training trial, the rat was placed in the center of the circular platform to freely explore for 240 sec. When the rat entered the goal box sooner than 240 sec, it was permitted to stay for 30 sec. If the rat did not enter the box within 240 sec, it was placed into the goal box for 60 sec to become familiarized with it. The platform was surrounded by a curtain that had different visual cues for spatial orientation. On the fifth day, the formal trial was performed, and each rat's movements were recorded over 240 sec. The time that the rat spent finding the goal box (escape time) was measured.

### Sucrose preference test

The sucrose preference test is used to assess anhedonia.^32^ For 4 days, the drinking water was removed and replaced by two identical bottles, one filled with water and the other containing 1% sucrose solution. The position of the bottles was exchanged every 6 h. The formal test was performed on the fifth day. The sucrose preference rate (SPR) was calculated as the ratio of consumed sucrose solution to the total volume of liquid consumed. A lower SPR is assumed to represent anhedonia.

### Forced swim test

The forced swim test is used as a measure of depression-like behavior.^32^ Each rat was placed into a Plexiglas bucket (diameter, 30 cm; height, 45 cm) filled to a height of 30 cm with 22°C–25°C water. The duration that the rat remained immobile during the last 4 min of a 6-min trial test was calculated. Immobility was defined as no active behavior, such as swimming, climbing, or diving.

### Immunofluorescence staining

Rats were anesthetized with 5% isoflurane and transcardially perfused with 0.01 M of phosphate-buffered saline (PBS) and 4% paraformaldehyde (PFA; pH 7.4). After 48 h of fixation with 4% PFA, brains were dehydrated with a 30% sucrose solution at 4°C for 5 days and then cut into 20-μm-thick coronal slices with a cryostat. Slices were rinsed with 0.01 M of PBS, blocked with 5% bovine serum albumin for 1 h at room temperature, and then incubated with rabbit anti-Iba1 (ionized calcium-binding adaptor protein 1) primary antibody (1:500; Wako Chemicals, Richmond, VA) overnight at 4°C.

Slices were rinsed and incubated with secondary antibody (rat antirabbit, 1:500; Invitrogen, Carlsbad, CA) for 1 h at room temperature. Finally, stained slices were examined under a fluorescence microscope (Eclipse TE2000-E, Nikon, Tokyo, Japan). Three sections per rat were viewed and photographed by an investigator and analyzed quantitatively. Iba1-positive cells were counted in three ipsilateral ROIs (3 fields per section × 3 sections per rat) with ImageJ software (NIH, Bethesda, MD). To correlate pathology with MRI findings, the fields selected for the Iba1 staining analyses were evenly distributed within each of the corresponding MRI ROIs. The number of rats with successful staining in each group was as follows: sham group (*n* = 4); mild TBI group (*n* = 3); moderate TBI group (*n* = 6); and severe TBI group (*n* = 6).

### Statistical analysis

All data are shown as mean ± standard deviation. The first analysis involved comparison of males and females in the APTw signals at each time point in each CCI group. To explore for possible sex differences and considering the small sample size for each sex, we applied a less-conservative approach by using the independent-sample *t*-test without correction for multiple comparisons so as to reduce the chance of a false-negative result. Except for APTw in the ipsilateral thalamus at 1 day, there were no significant sex differences (*p* > 0.05). Therefore, we combined males and females in all subsequent analyses. The main analysis then used one-way analysis of variance (ANOVA) followed by Tukey's *post hoc* test for multiple comparisons among the sham and three CCI groups at each time point. The second analysis involved interrogation of the relationships between regional Iba1-positive cells and regional APTw and between neurobehavior outcomes and regional APTw MRI measurements. For this analysis, we used Pearson's correlation analysis. These analyses were performed with SPSS software (version 16.0; SPSS, Inc., Chicago, IL). *P* < 0.05 was considered statistically significant.

## Results

### Time course of multi-parametric magnetic resonance imaging features in different regions of interest of controlled cortical impact rats

**FIG. 1.**
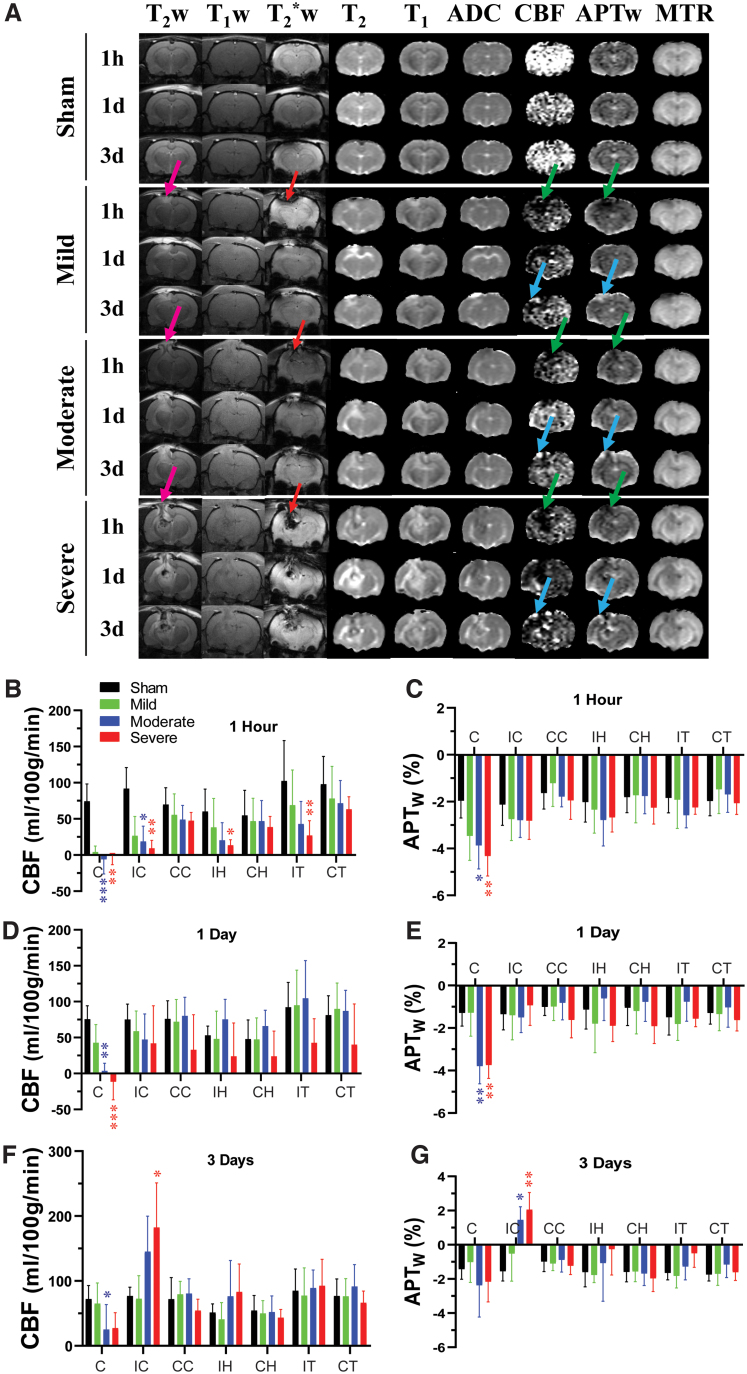
Temporal and spatial changes of multi-parametric MRI features in different ROIs in rats after CCI. (A) Representative multi-parametric MRI images in a rat at 1 h, 1 day, and 3 days post-CCI. Hemorrhage was observed in T_2_*w (red arrows) and T_2_w (pink arrows) scans. Note that susceptibility artifacts appeared in the contralateral hemisphere as well. The APTw and CBF signals in the core decreased markedly after CCI, especially at 1 h post-injury (green arrows). At 3 days post-CCI, the low APTw and CBF signals increased substantially, especially in the ipsilateral cortex (blue arrows). Quantitative analysis of CBF in seven ROIs at 1 h (B), 1 day (D), and 3 days (F) post-CCI. Quantitative analysis of APTw signals in different ROIs at 1 h (C), 1 day (E), and 3 days (G) post-CCI. *p < 0.05, **p < 0.01, ***p < 0.001 versus the sham group. These ROIs included the core (C), ipsi- and contralateral cortex (IC, CC), ipsi- and contralateral hippocampus (IH, CH), and ipsi- and contralateral thalamus (IT, CT). The display windows are T_2_ (0–100 ms), T_1_ (0.5–2.5 sec), ADC (0–2 × 10^–9^ m^2^/sec), blood flow (0–150 mL/100 g/min), APTw (−6% to 6% of the bulk water signal intensity), and MTR at 2 kHz (0–50% of the bulk water signal intensity).

**FIG. 2.**
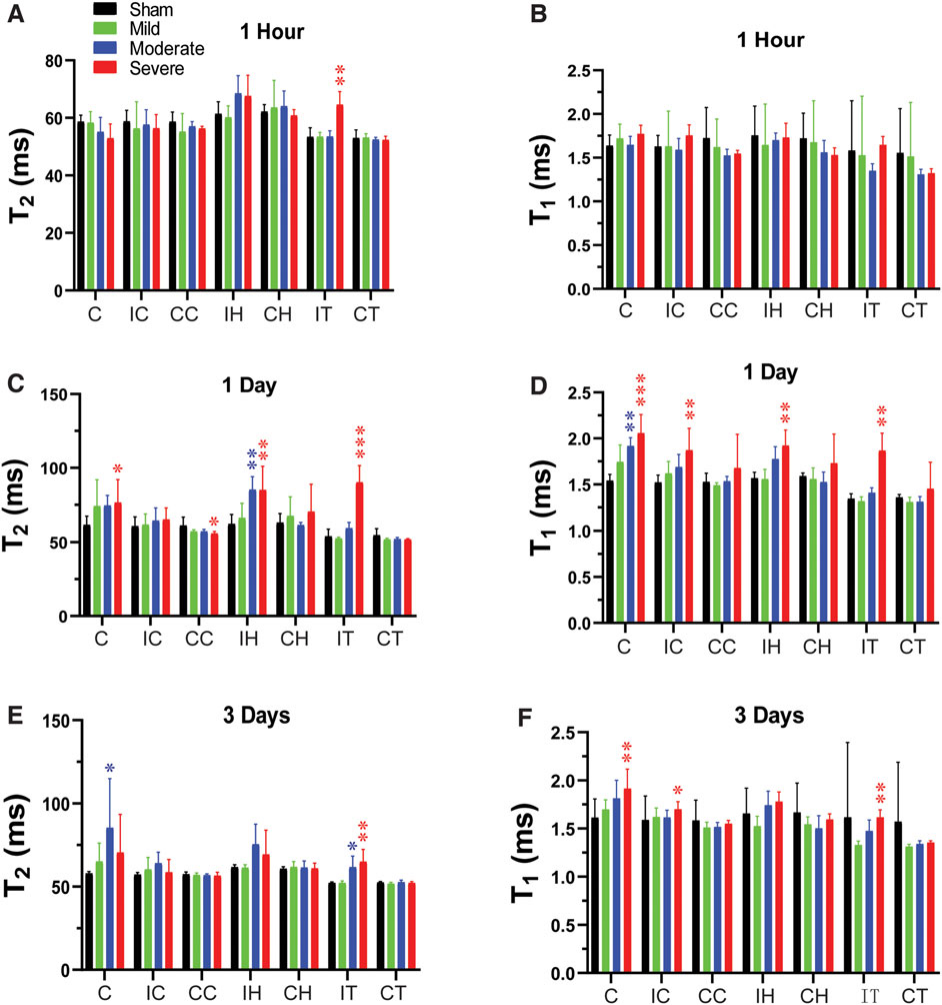
Temporal and spatial changes of T_2_ and T_1_ in different ROIs in rats after CCI. Quantitative analysis of T_2_ and T_1_ in seven ROIs at 1 h (A,B), 1 day (C,D), and 3 days (E,F) after different degrees of TBI. *p < 0.05, **p < 0.01, ***p < 0.001 versus the sham group. C, core; CC, contralateral cortex; CCI, controlled cortical impact; CH, contralateral hippocampus; CT, contralateral thalamus; IC, ipsilateral cortex; IH, ipsilateral hippocampus; IT, ipsilateral thalamus; ROI, region of interest; TBI, traumatic brain injury.

**FIG. 3.**
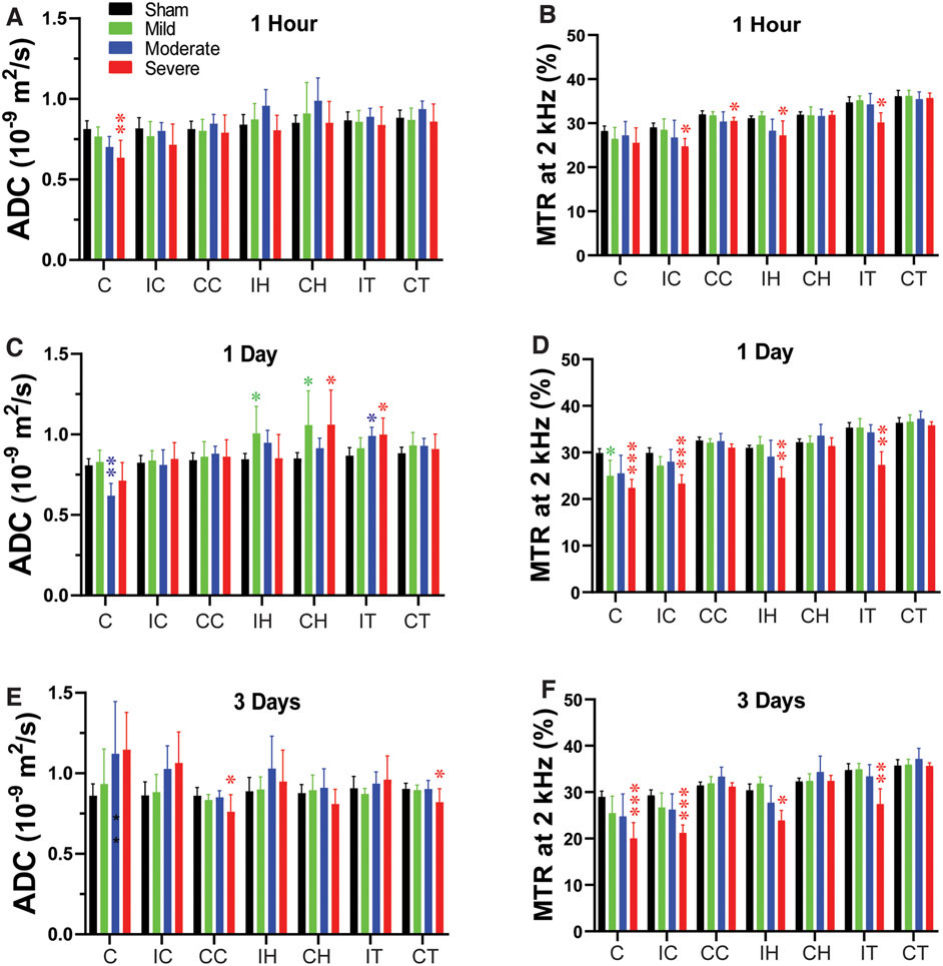
Temporal and spatial changes of ADC and MTR in different ROIs in rats after CCI. Quantitative analysis of ADC and MTR in seven ROIs at 1 h (A,B), 1 day (C,D), and 3 days (E,F) after different degrees of TBI. *p < 0.05, **p < 0.01, ***p < 0.001 versus the sham group. ADC, apparent diffusion constant; C, core; CC, contralateral cortex; CH, contralateral hippocampus; CT, contralateral thalamus; IC, ipsilateral cortex; IH, ipsilateral hippocampus; IT, ipsilateral thalamus; MTR, magnetization transfer ratio; TBI, traumatic brain injury.

**FIG. 4.**
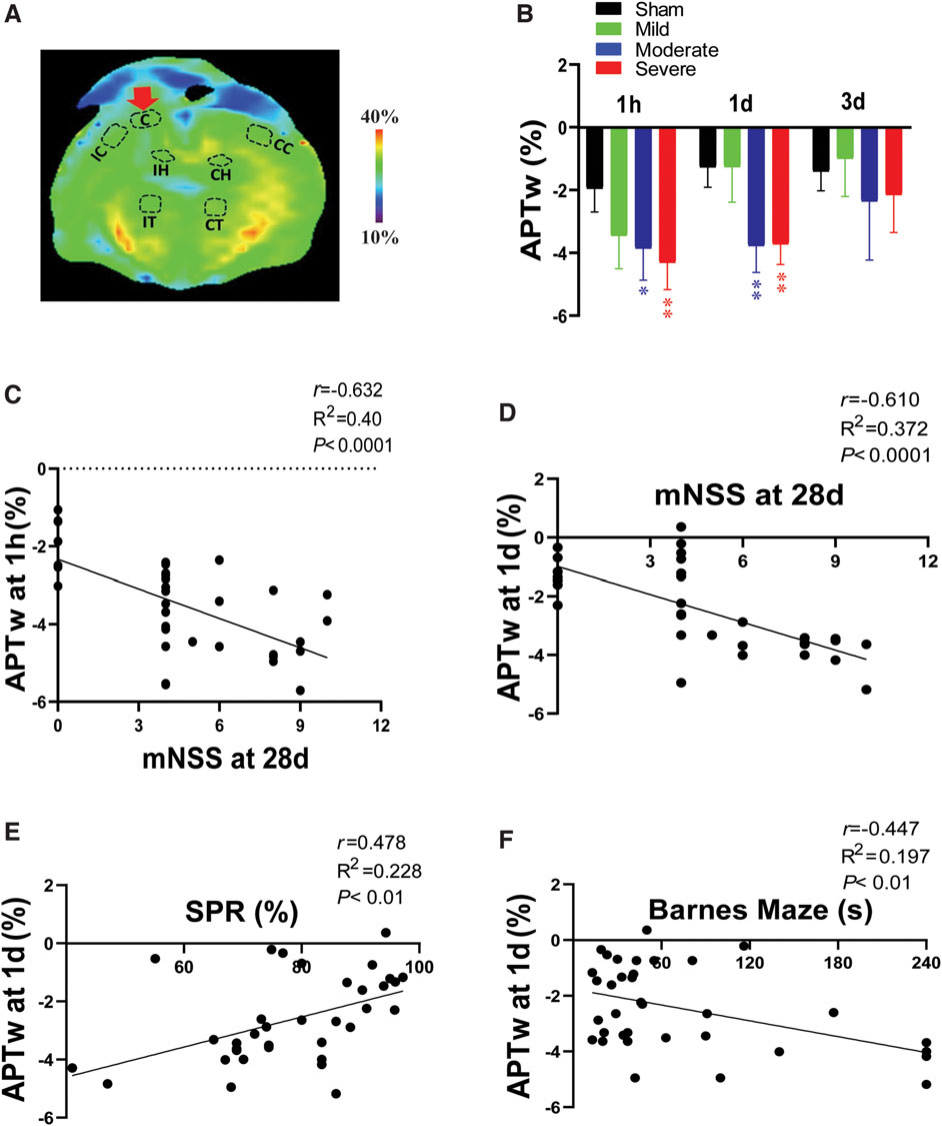
APTw signal in the injury core and its correlation with long-term disorders after TBI. (A) Example of a core region of interest on a color MTR image acquired from a female rat with moderate TBI at 3 days post-injury. (B) Quantitative analysis of APTw signal at 1 h, 1 day, and 3 days post-TBI. *p < 0.05, **p < 0.01 versus the sham group. (C,D) Correlation between APTw signal at 1 h (C) or 1 day (D) post-TBI and neurological score at 28 days post-TBI, as measured by the mNSS. (E,F) Correlation between APTw signal at 1 day post-TBI and sucrose preference rate (E) and time spent finding the goal box in the Barnes maze test (F) at 28 days after TBI. C, core; CC, contralateral cortex; CH, contralateral hippocampus; CT, contralateral thalamus; IC, ipsilateral cortex; IH, ipsilateral hippocampus; IT, ipsilateral thalamus; mNSS, modified neurological severity score; MTR, magnetization transfer ratio; SPR, sucrose preference rate; TBI, traumatic brain injury.

High-resolution MRI images (T_2_w and T_2_*w) showed primary injury (such as hemorrhage) on the impacted site (Fig. 1A). TBI contusion injury volumes at 28 days post-TBI were 0.0 ± 0.0, 37.6 ± 14.4, and 60.1 ± 14.1 mm^3^ (*F*_2, 25_ = 62.40, *p* < 0.0001) for mild/moderate/severe TBI groups, respectively. Quantitative multi-parametric MRI assessment results in different ROIs at 1 h, 1 day, and 3 days after TBI injury are shown in Figures 1–3. ROIs included the injury core, perilesion cortex, hippocampus, and thalamus ipsilateral to the injury and at the equivalent contralateral regions (Fig. 4A). At 1 h post-CCI, no significant change was observed for the mild group. However, the other groups exhibited significant changes in CBF (Fig. 1B), APTw (Fig. 1C), T_2_ (Fig. 2A), ADC (Fig. 3A), and MTR (Fig. 3B) compared to those of the sham group.

Specifically, in the moderate group, CBF and APTw were significantly reduced in the core, and CBF was reduced in the perilesion cortex. In the severe group, CBF was significantly reduced in all ipsilateral sites (Fig. 1A). APTw and ADC signals were significantly decreased only in the core at this early time, and the MTR signal was significantly decreased in the perilesion and contralateral cortex and in the ipsilateral hippocampus and thalamus.

At 1 day post-CCI, the mild group exhibited a significant decrease in MTR (Fig. 3D) in the core and significant increases in ADC (Fig. 3C) in the ipsi- and contralateral hippocampus. For the moderate group, CBF (Fig. 1D) and APTw (Fig. 1E) remained reduced in the core and were accompanied by a significant decrease in ADC (Fig. 3C). However, T_1_ (Fig. 2D) was significantly increased only in the core of the moderate group, whereas T_2_ (Fig. 2C) was significantly increased in the ipsilateral hippocampus. Similarly, in the severe group, CBF (Fig. 1D) and APTw (Fig. 1E) remained reduced in the core, whereas the increase in T_1_ (Fig. 2D) and decrease in MTR (Fig. 3D) expanded to all ipsilateral sites.

By day 3 after CCI, no noticeable differences were detected between the sham and mild groups. In the moderate group, the CBF (Fig. 1F) remained significantly decreased in the core, but T_2_ was increased in the core (Fig. 2E). Interestingly, APTw and CBF hyperintensities were clearly visible in the ipsilateral sites (Fig. 1A). Quantification showed that APTw signal (Fig. 1G) was markedly increased in the perilesion cortex of the moderate and severe groups, and CBF (Fig. 1F) was significantly increased in the perilesion cortex of the severe group. The APTw signal in the ipsilateral hippocampus of the severe group increased from 1 h to day 3, but the level at 3 days was not significantly different from that in the sham group. Moreover, the T_1_ (Fig. 2F) signal was increased in all ipsilateral regions except for the hippocampus, and MTR (Fig. 3F) decreased in all ipsilateral regions. Thus, different MRI signals have unique responses to TBI that vary with a given ROI, likely reflecting different aspects of dynamic pathophysiological processes occurring across brain regions.

### Correlation of amide proton transfer-weighted signal in the core area with long-term disorders after TBI

APTw signal in the injury core decreased acutely after CCI in a manner dependent on severity of the impactor displacement (Fig. 4B). Compared to the sham group, moderate and severe groups displayed significantly decreased APTw at 1 h and 1 day post-CCI. Additionally, APTw at 1 h and 1 day negatively correlated with the mNSS measured 28 days after CCI (Fig. 4C,D). Moreover, APTw at 1 day post-CCI showed a positive correlation with the preference for sucrose solution and a negative correlation with escape time in the Barnes maze test (Fig. 4E,F; Supplementary Table S1). These findings indicate that early APTw signals in the injury core are associated with the severity of CCI and that the change at 1 day post-TBI is significantly associated with neurological dysfunction, anhedonia, and memory decline 1 month later.

### Correlation of amide proton transfer-weighted signal in perilesion cortex with neuroinflammation and long-term outcomes after traumatic brain injury

**FIG. 5.**
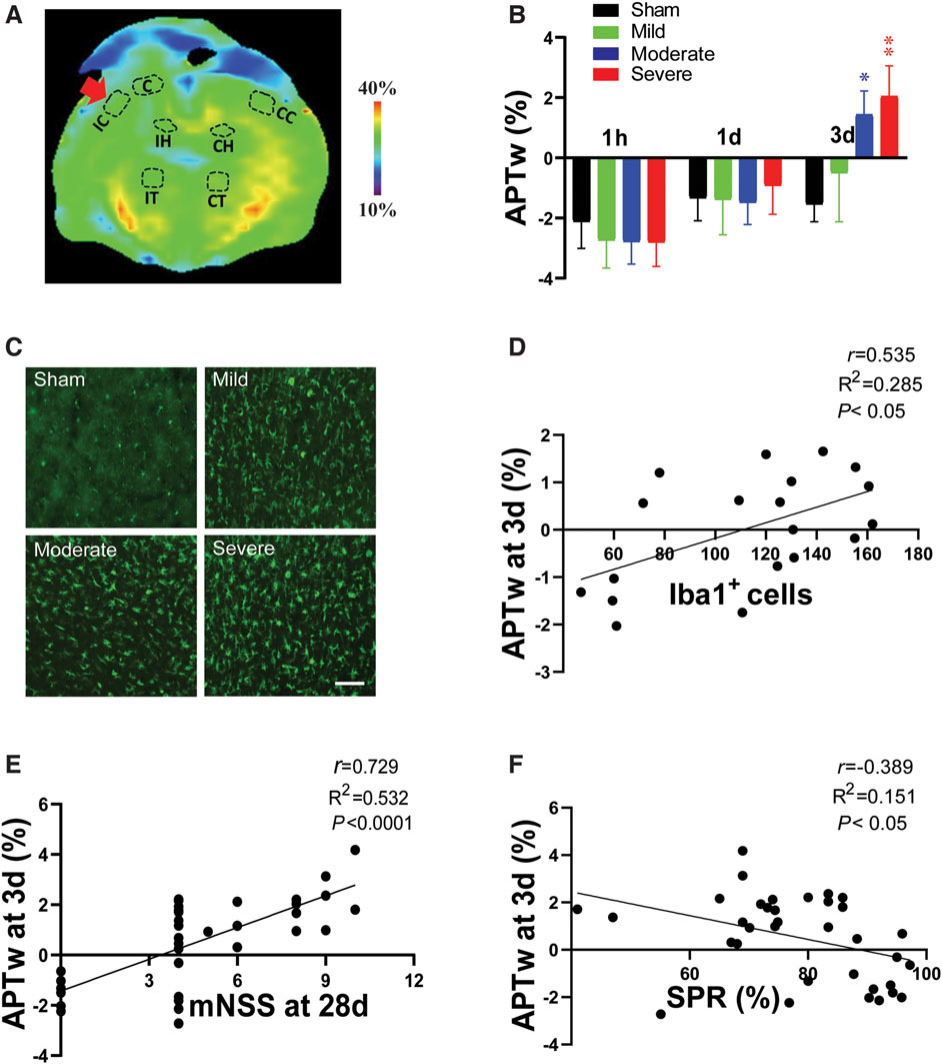
APTw signal in the perilesion cortex and its correlation to neuroinflammation and long-term outcomes after TBI. (A) Representative ipsilateral perilesion cortical (IC) region of interest on a color MTR image. (B) Quantitative analysis of APTw signal in the IC at 1 h, 1 day, and 3 days post-TBI. *p < 0.05, **p < 0.01 versus the sham group. (C) Representative images of Iba1 immunofluorescence staining in the IC on day 3 after TBI. Scale bar is 50 μm. The correlation between APTw signal at 3 days post-TBI and the number of Iba1-positive cells (D), neurological score (E), and sucrose preference rate (F) at 28 days post-TBI. C, core; CC, contralateral cortex; CH, contralateral hippocampus; CT, contralateral thalamus; IC, ipsilateral cortex; IH, ipsilateral hippocampus; IT, ipsilateral thalamus; mNSS, modified neurological severity score; MTR, magnetization transfer ratio; SPR, sucrose preference rate; TBI, traumatic brain injury.

APTw signal in the perilesion cortex lateral to the core injury gradually increased after CCI relative to that in the sham group, with significant increases in the moderate and severe groups at 3 days (Fig. 5B). The number of Iba1-positive cells was markedly higher in the perilesion cortex of the CCI groups than in that of the sham group at 3 days (Fig. 5C; also Supplementary Fig. S1) and showed a positive correlation with the APTw signal at 3 days (Fig. 5C,D; Supplementary Table S5). Further, APTw at 3 days positively correlated with the mNSS and negatively correlated with the SPR 1 month after CCI (Fig. 5E,F; Supplementary Table S2). Notably, these correlations in the perilesion cortex were directionally opposite to APTw in the injury core at 1 day. These data indicate that the APTw signal in the perilesion cortex at 3 days post-TBI is closely related to the neuroinflammatory response and associated with long-term neurological dysfunction and anhedonia.

### Correlation of amide proton transfer-weighted in the hippocampus with long-term disorders after traumatic brain injury

**FIG. 6.**
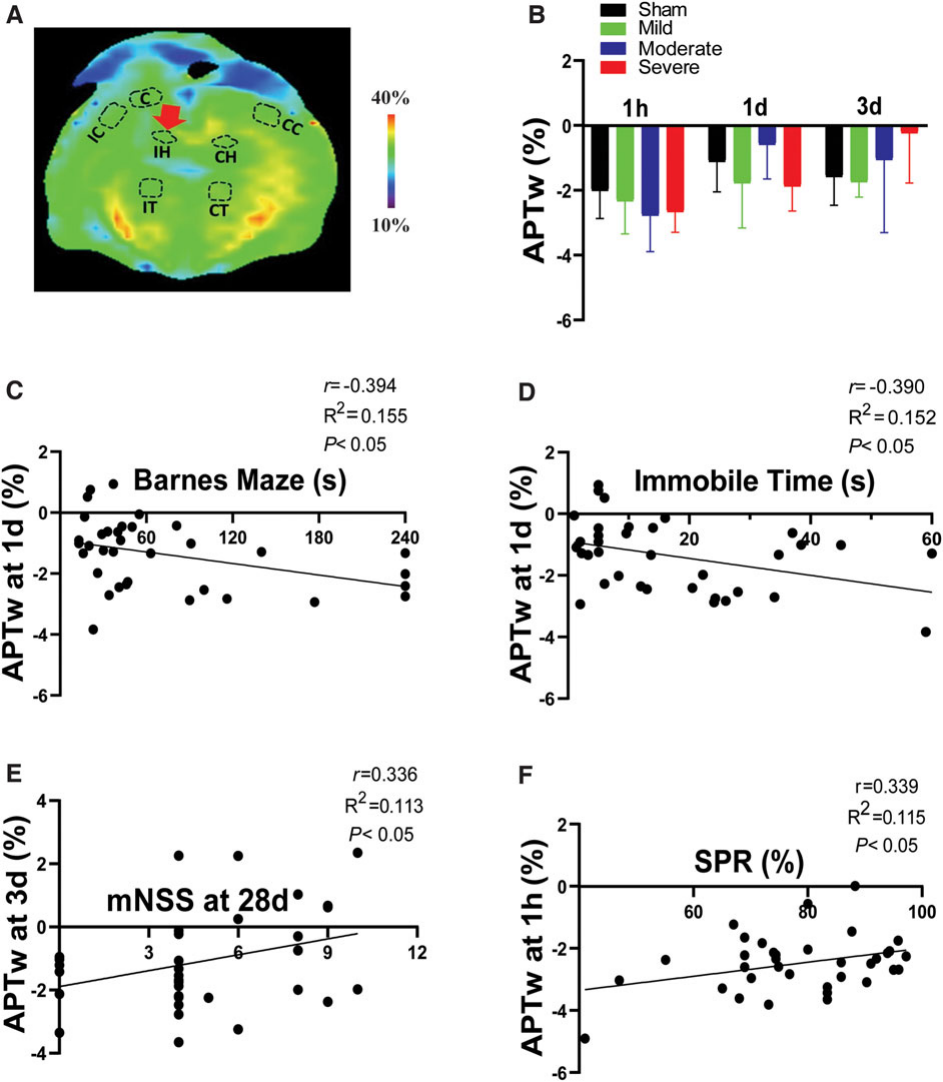
APTw signal in the ipsilateral hippocampus (IH) and its correlation with long-term outcomes after TBI. (A) Representative IH region of interest on a color MTR image. (B) Quantitative analysis of APTw signal at 1 h, 1 day, and 3 days post-TBI. (C,D) Correlation between APTw signal at 1 day post-TBI and time spent finding the goal box in the Barnes maze test (C) or immobile time in the forced swim test (D) at 28 days post-TBI. (E) Correlation between APTw signal at 3 days post-TBI and neurological score on day 28 after TBI. (F) Correlation between APTw signal at 1 h post-TBI and sucrose preference rate on day 28 after TBI. C, core; CC, contralateral cortex; CH, contralateral hippocampus; CT, contralateral thalamus; IC, ipsilateral cortex; IH, ipsilateral hippocampus; IT, ipsilateral thalamus; mNSS, modified neurological severity score; MTR, magnetization transfer ratio; SPR, sucrose preference rate; TBI, traumatic brain injury.

Mean values of APTw in the ipsilateral hippocampus displayed a graded increase with increasing CCI severity, but the variability was large and none of the mean values differed significantly from the sham group (Fig. 6B). Although the number of Iba1-positive cells in the hippocampus increased in a graded fashion with increasing CCI severity (Supplementary Fig. S1), the hippocampal APTw at 3 days did not correlate with Iba1-positive cell counts in hippocampus at 3 days, whereas MTR did (Supplementary Table S5). Nevertheless, the APTw signal at 1 day negatively correlated with the escape time in the Barnes maze test and immobilization time in the forced swim test (Fig. 6C,D). Further, APTw at 3 days post-CCI positively correlated with the mNSS after 28 days, and APTw at 1 h had a positive correlation with SPR at 28 days (Fig. 6E,F; Supplementary Table S3). These results indicate that the APTw signal may not be sensitive to injury severity or neuroinflammation in the hippocampus in the CCI model, but could serve as an early biomarker of long-term neurological dysfunction, memory decline, and anhedonia.

### Correlation of amide proton transfer-weighted in the thalamus with neuroinflammation and long-term disorders after traumatic brain injury

**FIG. 7.**
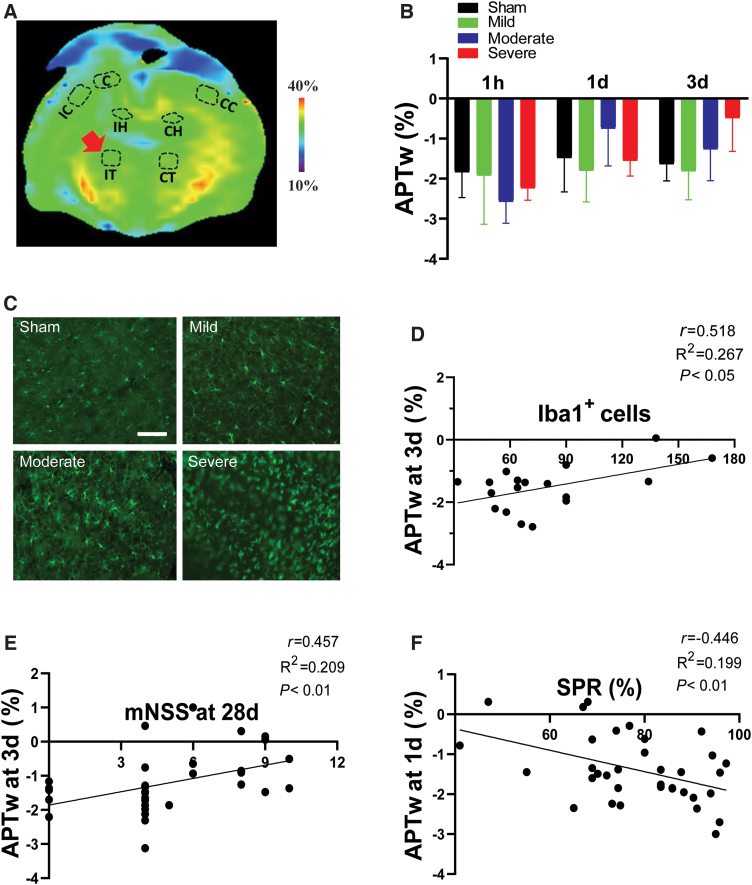
APTw signal in the ipsilateral thalamus (IT) and its correlation with neuroinflammation and long-term outcomes after TBI. (A) Representative IT region of interest on a color MTR image. (B) Quantitative analysis of APTw signal in the IT at 1 h, 1 day, and 3 days post-TBI. (C) Representative images of Iba1 immunofluorescence staining in the IT at 3 days post-TBI. Scare bar is 50 μm. (D) Correlation between APTw signal at 3 days post-TBI and the number of Iba1-positive cells at 3 days post-TBI or (E) neurological score on day 28 post-TBI. (F) Correlation between APTw signal at 1 day post-TBI and sucrose preference rate on day 28 after TBI. C, core; CC, contralateral cortex; CH, contralateral hippocampus; CT, contralateral thalamus; IC, ipsilateral cortex; IH, ipsilateral hippocampus; IT, ipsilateral thalamus; mNSS, modified neurological severity score; MTR, magnetization transfer ratio; SPR, sucrose preference rate; TBI, traumatic brain injury.

The number of Iba1-positive cells in the ipsilateral thalamus also increased, especially in the moderate and severe groups (Fig. 7C). The APTw signals in the ipsilateral thalamus showed a non-significant increase from 1 h to 3 days post-CCI, especially in the severe group (Fig. 7B). This trend was sufficient to provide a spread of APTw data such that it correlated positively with Iba staining (Fig. 7D; Supplementary Table S5). APTw at 3 days also correlated positively with the mNSS at 28 days (Fig. 7E), but correlated negatively with SPR at 1 day post-TBI (Fig. 7F; Supplementary Table S4). These data suggest that the APTw signal in the thalamus is a potential marker of neuroinflammation and might be useful for predicting long-term neurological dysfunction and depression.

## Discussion

In this study, we used noninvasive multi-parametric MRI techniques to evaluate the spatiotemporal evolution of different degrees of TBI damage and explored the capacity of APTw imaging to detect neuroinflammation and predict long-term outcomes. The results showed that all MRI signals assessed in this study were sensitive to TBI severity. Notably, APTw signals in the perilesion cortex of the moderate and severe groups were markedly increased at 3 days post-TBI, which can be visualized by eye, and values in the perilesion cortex and thalamus positively correlated with activated microglia at that time. Early APTw signals in the ipsilateral sites were closely related to long-term sequalae, including neurological dysfunction, memory decline, and depression. Thus, APTw imaging at 3 days after injury could be valuable for identifying neuroinflammation and predicting long-term outcomes.

The initial pathophysiological changes after CCI included reductions in CBF,^29^ which, if sufficiently severe, can lead to anaerobic glycolysis, lactic acid accumulation, altered ionic homeostasis, increased membrane permeability, and brain edema. Secondary injury from TBI is mainly attributed to excitotoxicity, neuroinflammation, persistent ischemic or hemorrhagic damage, and brain edema.^1,33,34^ Our past APTw imaging work with a single CCI severity was consistent with an ischemic core surrounded by an inflammatory region.^28,29^ The APT imaging technique can report on tissue pH or concentrations of endogenous mobile proteins and peptides.

By using different CCI severities, we confirmed significant decreases in CBF and APTw signals in the core of moderate and severe groups. Notably, this acute APTw hypointensity can be attributed to impaired energy metabolism and resultant tissue acidosis. The decrease in ADC in the injury core and the decreased MTR signal in multiple ipsilateral sites of the severe CCI group likely reflect cytotoxic edema.^35^ Moreover, we also found that APTw signals at 1 h post-CCI negatively correlated with the long-term deficits. These results suggest that CBF and APTw signals within 1 h in the core are sensitive biomarkers for the severity of the primary injury induced by CCI and could be beneficial as one prognostic indicator of long-term outcome.

Accumulating evidence indicates that neuroinflammation is critically important to secondary injury and influences long-term neurological deficits after TBI.^3,36–38^ However, few reliable, non-invasive MRI markers of neuroinflammation are available. Our previous work showed that high APTw signal intensity in the perilesion area at 3 days post-TBI was associated with increased glial activation.^29^ Here, we showed that the magnitude of the increase in APTw signal in the perilesion cortex at 3 days post-TBI above that in the sham group depended on the injury severity and, interestingly, correlated with the number of activated microglia, which release a variety of cytokines in the subacute stage of TBI.^3,34,37^

These results suggest that increased APTw signal in the perilesion cortex at 3 days may result from an increase in cellular proteins associated with the inflammatory process. This interpretation is based on other studies showing that APTw signal intensity positively correlates with cellular proteins in other neurological diseases, such as glioma^39,40^ and Parkinson's disease.^41^ Because the neuroinflammatory response affects long-term behavior and the APTw signal is associated with microglial activation, the APTw signal at 3 days appears to provide prognostic information about the long-term neurobehavior outcomes after TBI.

One limitation of this study is that only Iba1 staining was used as a surrogate of neuroinflammation. A more detailed analysis at 3 days would be useful to better discern the particular characteristics of the neuroinflammatory response that render the APTw hyperintensity. Thus, additional investigations on cellular molecular mechanisms linking the APTw signal with neuroinflammation are warranted. Another limitation is that the CCI model produces a primary focal injury whereas TBI is a diverse disorder. Whether APTw hyperintensity is as readily discernable in other TBI models with more diffuse injury remains to be determined. Nevertheless, the fact that we did observe some correlations of long-term behavior outcomes with APTw in the thalamus, which is distant from the primary injury, is encouraging. Finally, the sample size was relatively small in this study. Only the simple statistical methods (Pearson's correlation analysis and ANOVA) were utilized, instead of multi-level modeling statistics. A prospective study with a large sample size would be needed to determine the sensitivity, specificity, and predictive value of APTw relative to other MRI measurements.

In conclusion, our study illustrates the potential for APT imaging to assess the severity of injury and the level of inflammation at the acute stage of TBI. Because of the association of early APTw measurements with long-term behavioral outcome deficits, this non-invasive MRI parameter may be useful for stratifying patients in clinical trials and treatment regimens.

## Supplementary Material

Supplemental data

Supplemental data

Supplemental data

Supplemental data

Supplemental data

Supplemental data

## Abbreviations Used

ADC: apparent diffusion constant
ANOVA: analysis of variance
APTw: amide proton transfer-weighted
CBF: cerebral blood flow
CCI: controlled cortical impact
Iba1: ionized calcium-binding adaptor protein 1
mNSS: modified neurological severity score
MRI: magnetic resonance imaging
MTR: magnetization transfer ratio
NA: number of averages
PBS: phosphate-buffered saline
PFA: paraformaldehyde
ROI: region of interest
SPR: sucrose preference rate
T: Tesla
TBI: traumatic brain injury
TE: echo time
TR: repetition time
