# Supplementary material for: Amide Proton Transfer-Weighted Magnetic Resonance Imaging for Detecting Severity and Predicting Outcome after Traumatic Brain Injury in Rats
Source: Neurotrauma Rep. 2022 Jul 15;3(1):261–75. doi: 10.1089/neur.2021.0064 (PMC9380886; doi:10.1089/neur.2021.0064)
Supplement: Supplemental data [file Supp_TableS1.pdf]

SUPPLEMENTARY MATERIAL

Amide proton transfer-weighted MRI for detecting severity and predicting outcome after traumatic brain injury in rats

Yinfeng Dong, Yanting Gu, Jianhua Lu, Jieru Wan, Shanshan Jiang, Raymond C. Koehler, Jian Wang, Jinyuan Zhou

Table S1. Correlation between MRI signals in the injury core and behavior tests

Parameters		Modified Neurologic Severity Score						Barnes Maze		Sucrose Preference		Forced Swim	
		1d		3d		28d							
		r	*P*	*r*	*P*	*r*	*P*	*r*	*P*	*r*	*P*	*r*	*P*
APTw	1h	**-0.694**	**0.000**	**-0.720**	**0.000**	**-0.632**	**0.000**	-0.324	0.058	0.302	0.078	0.037	0.831
	1d	**-0.716**	**0.000**	**-0.634**	**0.000**	**-0.610**	**0.000**	**-0.447**	**0.007**	**0.478**	**0.004**	-0.172	0.323
	3d	-0.167	0.337	-0.140	0.424	-0.281	0.102	-0.018	0.920	0.247	0.153	-0.020	0.907
MTR	1h	-0.157	0.368	-0.152	0.383	-0.267	0.121	-0.053	0.761	0.180	0.301	-0.160	0.360
	1d	**-0.559**	**0.000**	**-0.615**	**0.000**	**-0.615**	**0.000**	-0.287	0.094	0.221	0.201	0.014	0.938
	3d	**-0.571**	**0.000**	**-0.605**	**0.000**	**-0.618**	**0.000**	**-0.441**	**0.008**	0.212	0.222	-0.122	0.484
CBF	1h	**-0.833**	**0.000**	**-0.787**	**0.000**	**-0.673**	**0.000**	-0.296	0.089	**0.384**	**0.025**	-0.218	0.215
	1d	**-0.814**	**0.000**	**-0.786**	**0.000**	**-0.762**	**0.000**	-0.322	0.060	**0.361**	**0.033**	-0.126	0.472
	3d	**-0.393**	**0.020**	**-0.34**	**0.046**	**-0.48**	**0.003**	-0.178	0.307	0.297	0.083	-0.006	0.974
ADC	1h	**-0.569**	**0.000**	**-0.581**	**0.000**	**-0.652**	**0.000**	**-0.343**	**0.044**	**0.419**	**0.012**	-0.299	0.081
	1d	**-0.569**	**0.000**	**-0.449**	**0.007**	**-0.385**	**0.023**	0.034	0.845	**0.414**	**0.013**	0.085	0.628
	3d	**0.419**	**0.012**	**0.387**	**0.022**	**0.359**	**0.034**	**0.406**	**0.016**	-0.24	0.165	0.108	0.538
T_1	1h	0.059	0.738	0.109	0.533	**0.339**	**0.046**	0.031	0.861	0.097	0.580	0.163	0.348
	1d	**0.765**	**0.000**	**0.784**	**0.00**	**0.711**	**0.000**	0.237	0.170	**-0.453**	**0.006**	0.053	0.764
	3d	**0.445**	**0.007**	**0.438**	**0.008**	**0.511**	**0.002**	0.205	0.238	-0.317	0.063	0.215	0.214
T_2	1h	-0.320	0.061	-0.327	0.055	**-0.467**	**0.005**	-0.266	0.123	0.245	0.156	0.107	0.539
	1d	0.331	0.052	**0.395**	**0.019**	0.296	0.085	**0.388**	**0.021**	-0.152	0.383	-0.004	0.098
	3d	0.193	0.266	0.148	0.398	0.176	0.313	0.211	0.224	-0.148	0.395	0.055	0.752
