# Supplementary material for: Amide Proton Transfer-Weighted Magnetic Resonance Imaging for Detecting Severity and Predicting Outcome after Traumatic Brain Injury in Rats
Source: Neurotrauma Rep. 2022 Jul 15;3(1):261–75. doi: 10.1089/neur.2021.0064 (PMC9380886; doi:10.1089/neur.2021.0064)
Supplement: Supplemental data [file Supp_FigS1.pdf]

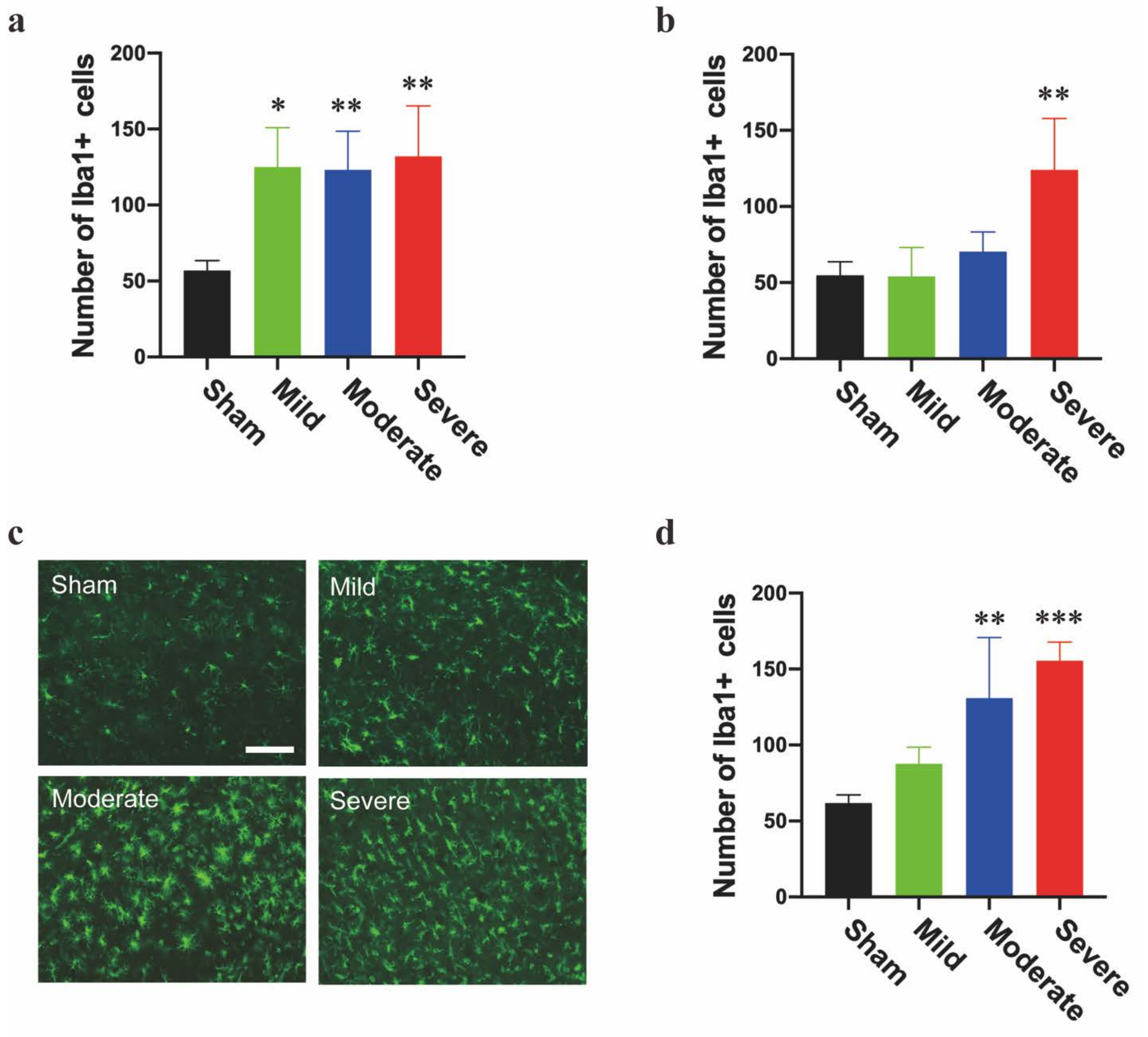

Supplementary Figure S1. Microglial counts in different ROIs at 3 days post-TBI. (a) The number of Iba1-positive cells in ipsilateral perilesion cortex. (b) The number of Iba1-positive cells in ipsilateral thalamus. (c) Representative images of Iba1 immunofluorescence staining in ipsilateral hippocampus and (d) the number of Iba1-positive cells in ipsilateral hippocampus. Scale bar is 50 μm. *$P < 0.05$, **$P < 0.01$, **$P < 0.001$ *vs.* sham group.
