# Supplementary material for: Amide Proton Transfer-Weighted Magnetic Resonance Imaging for Detecting Severity and Predicting Outcome after Traumatic Brain Injury in Rats
Source: Neurotrauma Rep. 2022 Jul 15;3(1):261–75. doi: 10.1089/neur.2021.0064 (PMC9380886; doi:10.1089/neur.2021.0064)
Supplement: Supplemental data [file Supp_TableS5.pdf]

Table S5. Correlation between MRI signals in the regions of interest and the number of Iba1-positive cells

Parameters	Ipsilateral cortex		Ipsilateral hippocampus		Ipsilateral thalamus	
	r	*P*	*r*	*P*	*r*	*P*
APTw	**0.535**	**0.018**	0.046	0.861	**0.518**	**0.028**
MTR	-0.330	0.167	**-0.714**	**0.001**	-0.254	0.309
CBF	-0.052	0.832	0.166	0.524	0.065	0.797
ADC	**0.532**	**0.019**	0.024	0.926	0.666	0.003
T_1	**0.657**	**0.002**	0.462	0.062	**0.869**	**0.000**
T_2	0.357	0.133	0.227	0.381	**0.759**	**0.000**
