# Supplementary material for: Amide Proton Transfer-Weighted Magnetic Resonance Imaging for Detecting Severity and Predicting Outcome after Traumatic Brain Injury in Rats
Source: Neurotrauma Rep. 2022 Jul 15;3(1):261–75. doi: 10.1089/neur.2021.0064 (PMC9380886; doi:10.1089/neur.2021.0064)
Supplement: Supplemental data [file Supp_TableS2.pdf]

Table S2. Correlation between MRI signals in the perilesion ipsilateral cortex and behavior tests

Parameters		Modified Neurologic Severity Score						Barnes Maze		Sucrose Preference		Forced Swim	
		1d		3d		28d							
		r	*P*	*r*	*P*	*r*	*P*	*r*	*P*	*r*	*P*	*r*	*P*
APTw	1h	**-0.457**	**0.006**	**-0.418**	**0.012**	-0.257	0.137	-0.275	0.110	0.122	0.484	0.088	0.614
	1d	0.005	0.978	0.095	0.589	0.189	0.277	-0.108	0.538	0.071	0.684	-0.202	0.245
	3d	**0.697**	**0.000**	**0.669**	**0.000**	**0.729**	**0.000**	0.306	0.074	**-0.389**	**0.021**	0.023	0.896
MTR	1h	-0.298	0.082	**-0.344**	**0.043**	**-0.473**	**0.004**	-0.269	0.118	0.227	0.190	-0.002	0.991
	1d	**-0.528**	**0.001**	**-0.581**	**0.000**	**-0.703**	**0.000**	**-0.482**	**0.003**	0.127	0.467	-0.014	0.936
	3d	**-0.569**	**0.000**	**-0.678**	**0.000**	**-0.699**	**0.000**	**-0.414**	**0.013**	0.167	0.337	-0.023	0.895
CBF	1h	**-0.730**	**0.000**	**-0.718**	**0.000**	**-0.681**	**0.000**	**-0.398**	**0.020**	0.329	0.058	-0.147	0.407
	1d	**-0.387**	**0.022**	**-0.357**	**0.035**	-0.313	0.067	-0.142	0.417	0.122	0.484	-0.178	0.305
	3d	**0.578**	**0.000**	**0.572**	**0.000**	**0.649**	**0.000**	**0.397**	**0.018**	-0.334	0.050	0.277	0.108
ADC	1h	-0.289	0.093	-0.330	0.053	**-0.424**	**0.011**	-0.312	0.068	0.102	0.559	-0.291	0.089
	1d	-0.068	0.699	-0.007	0.967	0.166	0.339	-0.061	0.730	0.190	0.275	-0.017	0.921
	3d	**0.496**	**0.002**	**0.497**	**0.002**	**0.368**	**0.030**	**0.380**	**0.024**	-0.317	0.063	0.055	0.754
T_1	1h	-0.063	0.720	-0.006	0.971	0.203	0.243	0.040	0.817	0.166	0.341	0.089	0.610
	1d	**0.582**	**0.000**	**0.590**	**0.000**	**0.601**	**0.000**	0.300	0.080	**-0.339**	**0.047**	-0.053	0.761
	3d	0.206	0.236	0.247	0.152	0.250	0.148	0.191	0.273	-0.145	0.405	-0.027	0.879
T_2	1h	-0.175	0.315	-0.198	0.253	-0.172	0.324	-0.179	0.303	-0.038	0.828	-0.011	0.949
	1d	0.203	0.243	0.210	0.226	0.144	0.409	**0.368**	**0.030**	-0.204	0.239	-0.120	0.491
	3d	0.206	0.235	0.125	0.473	0.019	0.912	0.304	0.076	-0.261	0.131	0.102	0.561
