# Supplementary material for: Amide Proton Transfer-Weighted Magnetic Resonance Imaging for Detecting Severity and Predicting Outcome after Traumatic Brain Injury in Rats
Source: Neurotrauma Rep. 2022 Jul 15;3(1):261–75. doi: 10.1089/neur.2021.0064 (PMC9380886; doi:10.1089/neur.2021.0064)
Supplement: Supplemental data [file Supp_TableS3.pdf]

Table S3. Correlation between MRI signals in the ipsilateral hippocampus and behavior tests

| Parameters | | Modified Neurologic Severity Score | | | | | | Barnes Maze | | Sucrose Preference | | Forced Swim | |
|---|---|---|---|---|---|---|---|---|---|---|---|---|---|
| | | 1d | | 3d | | 28d | | | | | | | |
| | | *r* | *P* | *r* | *P* | *r* | *P* | *r* | *P* | *r* | *P* | *r* | *P* |
| APTw | 1h | **-0.349** | **0.040** | **-0.364** | **0.031** | -0.196 | 0.260 | -0.222 | 0.200 | **0.339** | **0.047** | -0.001 | 0.996 |
| | 1d | -0.216 | 0.213 | -0.285 | 0.097 | -0.226 | 0.191 | **-0.394** | **0.019** | 0.072 | 0.679 | **0.390** | **0.021** |
| | 3d | 0.195 | 0.261 | 0.195 | 0.263 | **0.336** | **0.048** | -0.165 | 0.345 | -0.093 | 0.593 | -0.052 | 0.765 |
| MTR | 1h | **-0.564** | **0.000** | **-0.517** | **0.001** | **-0.487** | **0.003** | -0.169 | 0.332 | 0.317 | 0.063 | 0.057 | 0.746 |
| | 1d | **-0.433** | **0.009** | **-0.463** | **0.005** | **-0.626** | **0.000** | -0.189 | 0.278 | 0.110 | 0.531 | 0.158 | 0.366 |
| | 3d | **-0.464** | **0.005** | **-0.487** | **0.003** | **-0.618** | **0.000** | -0.268 | 0.120 | 0.181 | 0.298 | 0.007 | 0.969 |
| CBF | 1h | **-0.521** | **0.002** | **-0.497** | **0.003** | **-0.462** | **0.006** | -0.306 | 0.078 | 0.332 | 0.055 | -0.081 | 0.649 |
| | 1d | -0.130 | 0.456 | -0.211 | 0.223 | -0.253 | 0.143 | -0.062 | 0.723 | 0.011 | 0.951 | 0.012 | 0.946 |
| | 3d | 0.293 | 0.088 | 0.257 | 0.137 | 0.271 | 0.115 | 0.073 | 0.675 | -0.072 | 0.680 | 0.156 | 0.370 |
| ADC | 1h | 0.152 | 0.384 | 0.029 | 0.870 | -0.183 | 0.294 | -0.224 | 0.196 | -0.082 | 0.639 | -0.168 | 0.334 |
| | 1d | 0.083 | 0.633 | 0.074 | 0.674 | -0.091 | 0.604 | 0.199 | 0.253 | -0.143 | 0.412 | -0.042 | 0.813 |
| | 3d | 0.285 | 0.098 | 0.214 | 0.218 | 0.136 | 0.435 | 0.112 | 0.522 | -0.118 | 0.498 | -0.109 | 0.534 |
| $T_1$ | 1h | -0.124 | 0.478 | -0.147 | 0.400 | -0.002 | 0.990 | -0.103 | 0.556 | 0.205 | 0.237 | -0.017 | 0.925 |
| | 1d | **0.545** | **0.001** | **0.541** | **0.001** | **0.627** | **0.000** | 0.137 | 0.432 | -0.265 | 0.125 | -0.073 | 0.679 |
| | 3d | 0.171 | 0.326 | 0.143 | 0.412 | 0.259 | 0.133 | 0.143 | 0.411 | -0.211 | 0.224 | 0.036 | 0.838 |
| $T_2$ | 1h | **0.513** | **0.002** | **0.442** | **0.008** | **0.400** | **0.017** | 0.030 | 0.865 | -0.236 | 0.172 | -0.063 | 0.718 |
| | 1d | **0.560** | **0.000** | **0.520** | **0.001** | **0.483** | **0.003** | 0.260 | 0.131 | **-0.444** | **0.007** | 0.053 | 0.762 |
| | 3d | 0.298 | 0.083 | 0.202 | 0.245 | 0.152 | 0.382 | 0.254 | 0.140 | -0.232 | 0.181 | 0.226 | 0.192 |
