# Supplementary material for: Amide Proton Transfer-Weighted Magnetic Resonance Imaging for Detecting Severity and Predicting Outcome after Traumatic Brain Injury in Rats
Source: Neurotrauma Rep. 2022 Jul 15;3(1):261–75. doi: 10.1089/neur.2021.0064 (PMC9380886; doi:10.1089/neur.2021.0064)
Supplement: Supplemental data [file Supp_TableS4.pdf]

Table S4. Correlation between MRI signals in the ipsilateral thalamus and behavior tests

Parameters		Modified Neurologic Severity Score						Barnes Maze		Sucrose Preference		Forced Swim	
		1d		3d		28d							
		r	*P*	*r*	*P*	*r*	*P*	*r*	*P*	*r*	*P*	*r*	*P*
APTw	1h	-0.302	0.078	-0.290	0.091	-0.161	0.355	-0.065	0.712	0.205	0.238	-0.030	0.863
	1d	0.220	0.204	0.078	0.657	-0.026	0.881	-0.029	0.868	**-0.446**	**0.007**	0.255	0.139
	3d	**0.335**	**0.049**	**0.338**	**0.047**	**0.457**	**0.006**	0.205	0.238	-0.127	0.467	-0.021	0.906
MTR	1h	**-0.366**	**0.030**	**-0.492**	**0.003**	**-0.598**	**0.000**	-0.421	0.012	0.009	0.961	0.080	0.647
	1d	**-0.460**	**0.005**	**-0.558**	**0.001**	**-0.743**	**0.000**	**-0.419**	**0.012**	-0.025	0.888	0.088	0.614
	3d	**-0.446**	**0.007**	**-0.532**	**0.001**	**-0.694**	**0.000**	**-0.497**	**0.002**	-0.024	0.889	-0.038	0.826
CBF	1h	**-0.511**	**0.002**	**-0.545**	**0.001**	**-0.499**	**0.003**	-0.236	0.179	**0.394**	**0.021**	-0.123	0.487
	1d	-0.123	0.483	-0.217	0.210	**-0.392**	**0.020**	-0.048	0.786	0.059	0.735	0.097	0.578
	3d	0.099	0.572	0.123	0.482	0.046	0.794	-0.126	0.471	-0.018	0.917	0.075	0.667
ADC	1h	-0.126	0.471	-0.153	0.381	-0.267	0.121	0.093	0.596	0.109	0.532	-0.243	0.159
	1d	**0.527**	**0.001**	**0.501**	**0.002**	**0.522**	**0.001**	**0.482**	**0.003**	-0.196	0.259	0.257	0.136
	3d	0.271	0.115	0.276	0.108	0.232	0.181	**0.406**	**0.016**	-0.109	0.533	0.016	0.929
T_1	1h	-0.152	0.382	-0.094	0.593	0.069	0.692	0.021	0.906	0.325	0.057	0.056	0.748
	1d	**0.518**	**0.001**	**0.607**	**0.000**	**0.770**	**0.000**	**0.378**	**0.025**	-0.098	0.574	-0.068	0.699
	3d	-0.042	0.811	-0.034	0.846	0.048	0.786	0.022	0.898	-0.118	0.499	-0.065	0.711
T_2	1h	**0.446**	**0.007**	**0.591**	**0.000**	**0.682**	**0.000**	**0.556**	**0.001**	0.030	0.866	0.126	0.469
	1d	**0.570**	**0.000**	**0.661**	**0.000**	**0.799**	**0.000**	**0.493**	**0.003**	-0.130	0.457	0.051	0.772
	3d	**0.546**	**0.001**	**0.564**	**0.000**	**0.615**	**0.000**	0.188	0.280	-0.311	0.069	0.201	0.246
